# The Effects of *Lycium chinense*, *Cuscuta chinensis*, *Senna tora*, *Ophiopogon japonicus*, and *Dendrobium nobile* Decoction on a Dry Eye Mouse Model

**DOI:** 10.3390/medicina58081134

**Published:** 2022-08-21

**Authors:** Cheng-Chan Yang, Jia-Ying Chien, Yu-Yau Chou, Jhih-Wei Ciou, Shun-Ping Huang

**Affiliations:** 1 Department of Chinese Medicine, Hualien Tzu Chi Hospital, Hualien 970, Taiwan; 2 School of Postbaccalaureate Chinese Medicine, Tzu Chi University, Hualien 970, Taiwan; 3 Institute of Medical Science, Tzu Chi University, Hualien 970, Taiwan; 4 Department of Molecular Biology and Human Genetics, Tzu Chi University, Hualien 970, Taiwan; 5 Department of Ophthalmology, Taichung Tzu Chi Hospital, Taichung 472, Taiwan

**Keywords:** dry eye disease, lacrimal gland, *Lycium chinense*, *Cuscuta chinensis*, *Senna tora*, *Ophiopogon japonicu*, *Dendrobium nobile*, inflammation

## Abstract

*Background and objective*: Dry eye disease (DED) is a relatively common disorder associated with abnormal tear film and the ocular surface that causes ocular irritation, dryness, visual impairment, and damage to the cornea. DED is not a life-threatening disease but causes discomfort and multifactorial disorders in vision that affect daily life. It has been reported that all traditional medicinal plants exhibit anti-inflammatory effects on several diseases. We hypothesized that the decoction ameliorated ocular irritation and decreased cytokine expression in the cornea. This study aimed to investigate the molecular mechanisms of DED and discover a therapeutic strategy to reduce corneal inflammation. *Material and Methods*: We used a DED mouse model with extraorbital lacrimal gland (ELG) excision and treated the mice with a decoction of five traditional medicines: *Lycium chinense*, *Cuscuta chinensis*, *Senna tora*, *Ophiopogon japonicus*, and *Dendrobium nobile* for 3 months. The tear osmolarity and the ocular surface staining were evaluated as indicators of DED. Immunohistochemistry was used to detect the level of inflammation on the cornea. *Results*: After treatment with the decoction for three months, epithelial erosions and desquamation were reduced, the intact of corneal endothelium was maintained, and tear osmolarity was restored in the eyes. The IL-1β-associated inflammatory response was reduced in the cornea in the DED model. *Conclusions:* These data suggested that a mixture of traditional medicines might be a novel therapy to treat DED.

## 1. Introduction

Dry eye disease (DED) is a common ocular surface disease that leads to eye discomfort, ocular irritation, dryness, visual impairment, and corneal damage. DED is associated with an unstable tear film that progressively compromises vision and affects the daily activities of patients [1,2]. The pathogenesis of DED is complex and multifactorial and involves autoimmune disorders, infections, the use of contact lenses, hormonal changes, chronic inflammation, and environmental factors [3,4]. DED diagnosis depends on subjective symptoms and dry eye tests, including ocular surface staining, tear film breakup time (TFBUT), tear secretion volume measurement by Schirmer’s test, and tear osmolarity [5,6]. As DED is a common disease, it may affect quality of life worldwide. It is necessary to investigate the molecular mechanisms involved in DED and understand the pathogenesis of DED.

Traditional medicinal herbs have been used as therapeutic agents for millions of years to cure disease and explore potential health properties. Five different traditional medicinal plants (*Lycium chinense*, *Cuscuta chinensis*, *Senna tora*, *Ophiopogon japonicus*, and *Dendrobium nobile*) have been widely used to treat many diseases. Extracts of *Lycium chinense* induced anti-inflammatory responses by suppressing interleukin (IL)-1β and tumor necrosis factor (TNF)-α expression and lipopolysaccharide (LPS)-induced inflammation [7] and exerting antioxidant effects to treat Parkinson’s disease [8]; moreover, neuroprotective effects in retinal ischemia and reperfusion injury studies have been observed [9,10]. The extraction of *Cuscuta chinensis* has bioactive natural flavonoids and exerts antioxidant, anti-inflammatory, anticancer, and neuroprotective effects on an LPS-induced murine autoimmune model by reducing IL-6, TNF-α, and NF-κB levels [11,12]. The seed extracts of *Senna tora* exert biological activities, including anti-inflammatory and neuroprotective effects on LPS-induced RAW264.7 macrophages, hippocampal neurons, and retinal precursor cells by modulating the NF-κB pathway [13,14]. The root of Ophiopogon japonicus has been reported to have immunomodulatory and antioxidative effects on liver and lung injury [15,16,17] by suppressing the NF-κB pathway. Several useful chemical components extracted from Dendrobium nobile have antithrombotic and immunomodulatory effects on LPS-induced murine macrophage models and retinal cell models by inhibiting VEGP, COX-2, IL-6, and IL-1β production [18,19]. We hypothesized that a decoction of five traditional medicinal herbs may have therapeutic effects on DED.

It has been reported that many animal models of DED have been established to mimic the pathogenesis of DED, including monkey, rabbit, mouse, and rat models [20,21,22]. However, some genetically modified and spontaneous mouse models may not sufficiently mimic DED pathogenesis, such as damage to the ocular surface. Additionally, many models are difficult to implement in experiments. In this study, lacrimal gland excision was used to establish a DED model [23] to determine the molecular mechanism of the decoction. We confirmed that after exorbital lacrimal gland (ELG) excision, inflammatory responses in the epithelium and Bowman’s layer were decreased and corneal thickness was suppressed by decoction treatment. In the future, this decoction might be a novel treatment for DED.

## 2. Materials and Methods

### 2.1. Animals

Thirty-six C57BL/6J mice (8 weeks old) were purchased from the National Laboratory Animal Center and maintained in the Laboratory Animal Center at Tzu Chi University. This study was performed in accordance with the IACUC (No. 109011). Mice were housed under conventional conditions with a 12 h light and dark cycle and provided food and water ad libitum.

### 2.2. Lacrimal Gland Excision

**Figure 1:**
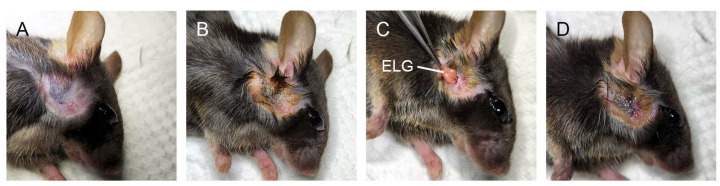
Extraorbital lacrimal gland (ELG) excision. (**A**) After anesthetization, the hair was shaved. (**B**) A 3 mm area anterior and ventral to the ear was cleansed with iodine tincture. (**C**) A 3 mm incision was made and the extraorbital lacrimal gland was excised. (**D**) The incision was sutured with 6-0 ophthalmic nylon thread and the incision was covered with ointment.

The mice were anesthetized by an intraperitoneal injection of 100 mg/kg ketamine (Health-Tech Pharmaceutical Co., Ltd., Taipei, Taiwan) and 10 mg/kg xylazine (Health-Tech Pharmaceutical Co., Ltd., Taiwan). Vidisic gel (Dr. Gerhard Mann Chem.-Pharm., Berlin, Germany) was dropped on the eye. After shaving the hair, a 3 mm area anterior and ventral to the ear was cleansed with iodine tincture, and an incision was made in the ear on the right side to expose the ELG with a stereoscopic microscope. The ELG was excised, and the incision was sutured with 6-0 ophthalmic nylon thread (Figure 1). An ointment was applied to the incision (0.3% tobramycin and 0.1% dexamethasone, TobraDex, Alcon, Puurs, Belgium).

### 2.3. Decoction Preparation

Eight grams of the dry seeds of *Lycium chinense*, 8 g of the dry seeds of *Cuscuta chinensis*, 8 g of the dry seeds of *Senna tora*, 8 g of the dry roots of *Ophiopogon japonicus*, and 8 g of the dry stems of *Dendrobium nobile* were crushed and mixed in 100 mL of drinking water. After being boiled in water, the decoction was isolated from the mixture.

### 2.4. Treatment with the Decoction

The concentrations of the herbs for human treatment are 20 g of five traditional medicines per day. The guide for dose conversion between animals and human follows the equivalent dose: Mouse equivalent dose (mg/kg) = (Dose to be converted)/(Mouse K_m_/Human K_m_). The average body weights of humans and mice are 63 kg and 25 g. The Km for humans and mice is 37 and 3, respectively [1]. The mouse equivalent dose (mg/kg) is 3909.6 mg/kg. The water intake per day in C57BL/6J mice is 7 mL [2]. The final concentration of the herbs of C57BL/6J mice is 13.96 mg/mL/day.

The decoction was diluted in a ratio of 28:1 in drinking water. The diluted decoction was given immediately after ELG excision. The decoction was administered for 3 months. The sham and ELG excision groups received normal drinking water during all experimental procedures.

### 2.5. Tear Osmolarity Measurement

Before ELG excision, mice were anesthetized by an intraperitoneal injection of 100 mg/kg ketamine and 10 mg/kg xylazine. Eye drops containing 0.5% Alcaine (Alcon, Puurs, Belgium) were administered before tear osmolarity measurement. Tear osmolarity was measured using an osmolarity system (I-Pen Vet Osmolarity, I-MED Pharma, Saint-Laurent, QC, Canada). After 3 months of decoction treatment, the tear osmolarity was measured after anesthesia.

### 2.6. Ocular Surface Staining

**Figure 2:**
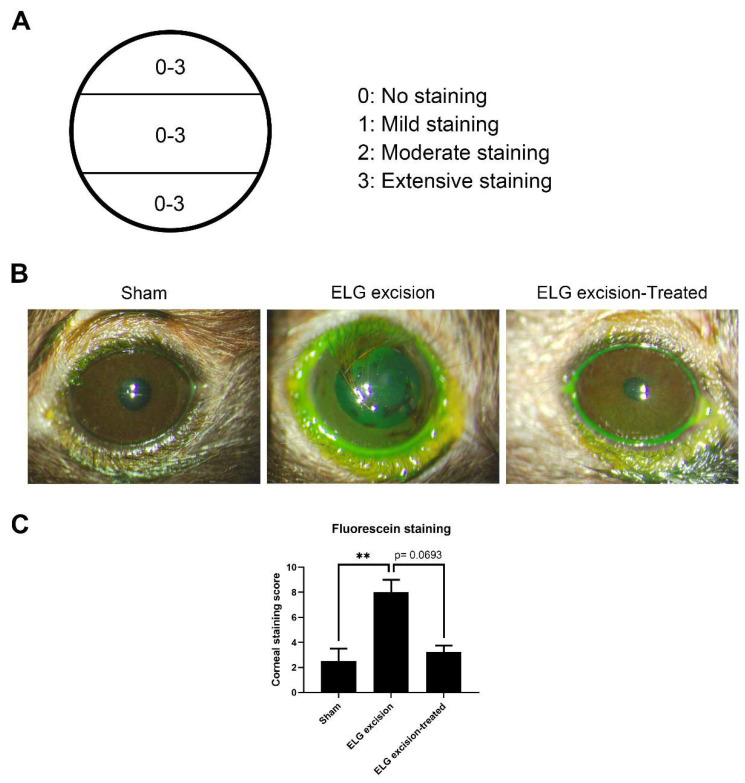
Fluorescein staining of the ocular surface. (**A**) Assessment of the corneal surface. (**B**) Decrease in punctate epithelial erosions on the cornea after decoction treatment. (**C**) The columns represent the defects in corneal staining, and the scores in the sham, ELG excision, and ELG excision-treated groups were 2.5 ± 1, 8 ± 1, and 3.25 ± 0.5, respectively. (n = 12, ** *p* < 0.001).

After 3 months of treatment, the mice were anesthetized by an intraperitoneal injection of 100 mg/kg ketamine and 10 mg/kg xylazine. A fluorescein sodium ophthalmic strip (1 mg/strip) (HUB Pharmaceuticals, Scottsdale, AZ, USA) was diluted in 300 μL of 0.9% normal saline to make the solution. Then, 20 μL of the solution was added dropwise to the conjunctival sac of each eye. The eyes were examined with a visual electrodiagnostic system (Espion, Diagnosys LLC, Gaithersburg, MA, USA). To assess corneal staining, we used the ocular surface score to evaluate damage to the cornea, and the cornea-divided horizon was assessed on a scale of 0 to 3 (total of 9 points) (Figure 2A) [24].

### 2.7. Histology

The cornea was excised and fixed in 4% paraformaldehyde (#43368, Alfa Aesar, Tewksbury, MA, USA) for 2 h at 25 °C. The tissue was embedded in OCT compounds (#4583, SAKURA, Torrance, CA, USA) and cut into 8 μm thick sections by a cryostat (Leica CM3050S, Deer Park, IL, USA). Hematoxylin (#1.09249.0500, Sigma, St. Louis, MO, USA) and eosin (#1.09844.1000, Merck, Darmstadt, Germany) staining was performed on each group. The stained sections were photographed by microscope (EVOS™ M5000 Imaging System, Invitrogen, Waltham, MA, USA). The detached corneal epithelial cells were calculated per section at 40× magnification (n = 12 in each group) and quantified with ImageJ software (version 1.8.0_172; U.S. National Institutes of Health, Bethesda, MD, USA, https://imagej.nih.gov/ij/).

### 2.8. Immunohistochemistry (IHC)

Frozen corneal sections were blocked with 1% BSA and labeled with IL-1β (1:200, Abcam) and IL-6 (1:500, Abcam) primary antibodies at 4 °C overnight. The sections were incubated with corresponding Alexa Fluor-conjugated secondary antibodies (1:100, Invitrogen, USA). Photographs were taken with a Zeiss LSM 900 confocal system (Zeiss, Oberkochen, Germany). At least six images per eye at 20× magnification were collected to quantify the number of positive cells in the cornea with ImageJ software.

### 2.9. Statistical Analysis

All the data are shown as the mean ± standard deviation (SD). Statistical analysis was performed by the Kruskal–Wallis test for comparisons between groups with GraphPad Prism 5 (GraphPad Software, La Jolla, CA, USA), and p values less than 0.05 were considered to indicate statistical significance.

## 3. Results

### 3.1. Decrease in Superficial Punctate Epithelial Erosions on the Corneas of ELG Excision Mice

Fluorescein sodium was used to confirm corneal epithelial damage and study dry eye [25,26]. To investigate ocular surface defects, fluorescein staining was used 3 months after ELG excision. Epithelial erosions in the cornea were ameliorated after 3 months of decoction treatment, and the numbers of puncta in the sham, ELG excision-only, and ELG excision-treated groups were 2.5 ± 1, 8 ± 1, and 3.25 ± 0.5, respectively. After 3 months of treatment with the decoction, the damage to the ocular surface was decreased (Figure 2). These results suggested that the decoction may rescue DED.

### 3.2. The Suppression of Tear Osmolarity after Decoction Treatment and ELG Excision

**Figure 3:**
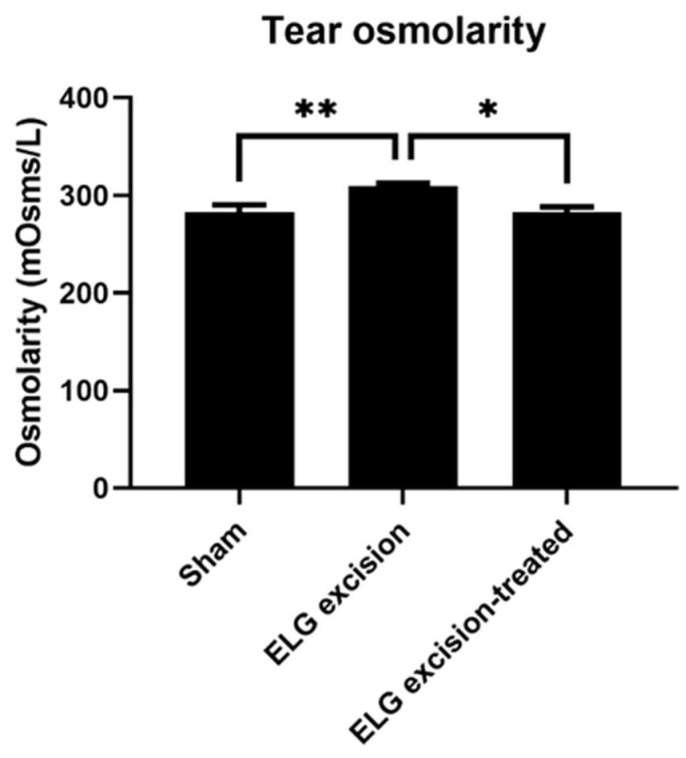
Tear osmolarity. Tear osmolarity was measured 3 months after ELG excision. The results in the sham, ELG excision, and ELG excision-treated groups were 282.8 ± 7.264, 309.5 ± 2.887, and 282.9 ± 5.146, respectively. (n = 12, * *p* < 0.05, ** *p* < 0.001).

Osmolarity measurement has superior diagnostic performance and is the best single metric to classify DED [27,28]. In the decoction treatment group, the osmolarity showed a significant reduction (Figure 3). The results in the sham, ELG excision, and ELG excision-treated groups were 282.8 ± 7.264, 309.5 ± 2.887, and 282.9 ± 5.146, respectively. These results confirmed the reduction in osmolarity by the decoction after ELG excision.

### 3.3. Decrease in Desquamation on the Corneal Epithelium after Decoction Treatment in the DED Model

**Figure 4:**
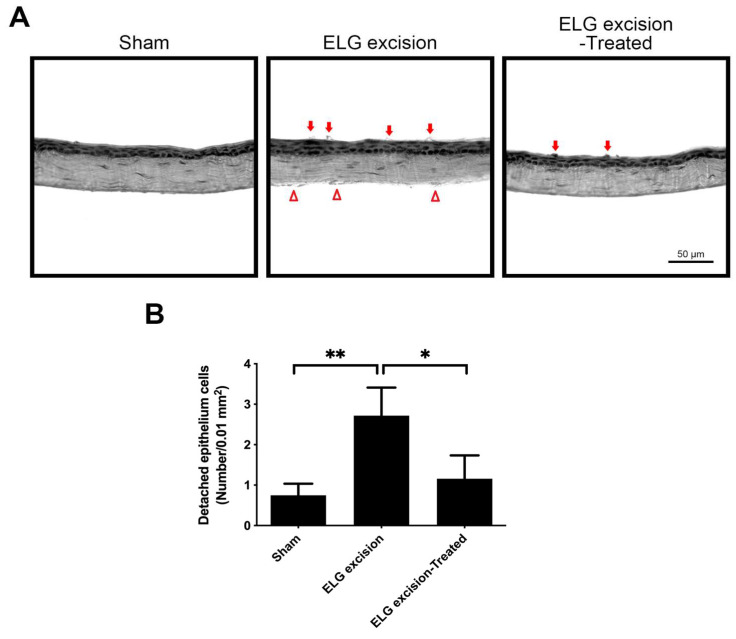
The effect of treatment with decoction on the desquamation of the corneal epithelium. (**A**) The corneal sections were stained with H&E after 3 months of decoction treatment. The red arrows indicate detached corneal epithelial cells. (Scale bar: 50 μm). The endothelial defects were observed in the cornea of ELG excision group (red triangles). (**B**) The quantitative data showed that the numbers of detached epithelial cells per 0.01 mm^2^ in sham, ELG excision, and ELG excision-treated groups were 0.75 ± 0.29, 2.72 ± 0.69, and 1.16 ± 0.52, respectively. (n = 12, * *p* < 0.05, ** *p* < 0.001).

The corneal sections were stained with H&E after being treated with the decoction for 3 months (Figure 4). The number of detached epithelial cells was reduced in the ELG excision-treated group (Figure 4A). The quantitative data showed that the numbers of detached epithelial cells per 0.01 mm^2^ in sham, ELG excision, and ELG excision-treated groups were 0.75 ± 0.29, 2.72 ± 0.69, and 1.16 ± 0.52, respectively (Figure 4B). The endothelial defects were observed in the corneas of the ELG excision group. In contrast, treatment with the decoction for 3 months might reduce the desquamation of corneal epithelium and maintain the corneal endothelium.

### 3.4. Reduced Inflammatory Responses after Decoction Treatment in the DED Model

**Figure 5:**
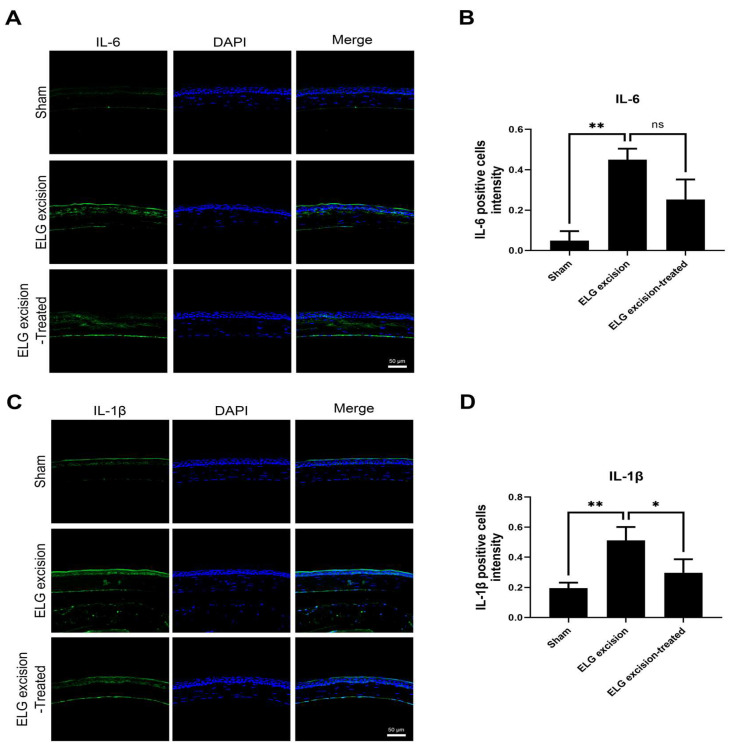
IHC analysis of IL-1β and IL-6 in the ocular surface. (**A**) Green fluorescence represents IL-1β-positive cells, and blue fluorescence represents the nuclei. (**B**) The columns indicate the intensity of green fluorescence in IL-1β-positive cells. (Scale bar: 50 μm, * *p* < 0.05, ** *p* < 0.001). (**C**) Green fluorescence represents IL-6-positive cells, and blue fluorescence represents the nuclei. (**D**) The columns indicate the intensity of green fluorescence in IL-6-positive cells. (n = 12, Scale bar: 50 μm, * *p* < 0.05, ns = 0.1884).

Previous studies have shown that innate immune system-mediated inflammation occurs in dry eye [29], leading to the expression of proinflammatory cytokines, including IL-1β and IL-6, on the ocular surface [30]. Immunohistochemical staining demonstrated suppression of IL-1β and IL-6 expression on the ocular surface in the decoction-treated group (Figure 5). The IL-1β levels in the sham, ELG excision, and ELG excision-treated groups were 0.1957 ± 0.036, 0.512 ± 0.088, and 0.298 ± 0.087, respectively. The numbers of IL-1β-positive cells were significantly decreased in the ELG excision-treated group (Figure 5A,B). The IL-6 intensities in the sham, ELG excision, and ELG excision-treated groups were 0.049 ± 0.0468, 0.4495 ± 0.054, and 0.252 ± 0.099, respectively. These results showed the reduction in IL-6 expression due to decoction treatment after ELG excision (Figure 5C,D). These data confirmed that the decoction may suppress inflammation in DED.

## 4. Discussion

Morphological and physiological changes were observed in a DED model, and there was an increase in tear osmolarity, defects in the ocular surface, and inflammatory responses in mice with ELG excision. DED is defined as a multifactorial disease of the ocular surface, and extrinsic and intrinsic factors trigger inflammation in dry eye. Allergic conjunctivitis and the environment induce autoimmune activation, leading to proinflammatory cytokine release in the ocular surface and exacerbating dry eye [31]. To modulate the inflammatory changes in dry eye, immune cells infiltrate the conjunctiva, cornea, and lacrimal glands and elevate tear cytokine levels in dry eye [32]. After desiccating stress on the corneal surface, ocular surface cells secrete inflammatory cytokines, especially TNF-α, IL-6, and IL-1β, triggering antigen presenting cell (APC) activation. APCs stimulate T cells, inducing further cytokine release, and interact with lymph nodes, leading to the upregulation of effector responses, an increase in immune cells on the ocular surface, and defects in corneal cells [33]. Indeed, inhibiting inflammation may rescue dry eye.

It has been reported that the compounds isolated from *Lycium chinense*, *Cuscuta chinensis*, *Senna tora*, *Ophiopogon japonicus*, and *Dendrobium nobile* have antioxidative, anti-inflammatory, and neuroprotective effects on several cell and animal disease models [34,35]. Aurantio-obtusin is an anthraquinone compound extracted from the seeds of *Senna tora* that suppressed NF-κB activation in an LPS-induced macrophage study [14]. Alkaloids isolated from *Dendrobium nobile* had neuroprotective effects on an LPS-induced BV2 microglial model by suppressing IL-1β [36]. Dihydro-N-caffeoyltyramine (DHCT) isolated from *Lycium chinense* decreased the NF-κB activity levels and antifungal effects of macrophage cells in PMA-mediated induction [1]. Quercetin and kaempferol isolated from *Cuscuta chinensis* was associated with increases in brain mitochondrial biogenesis and inhibited COX-2 protein expression in an atherosclerosis rabbit model and exercise mouse model [2,3,4]. Homoisoflavonoids from the tuberous roots of Ophiopogon japonicus suppressed apoptosis effects on an ischemia/reperfusion-induced myocardial mouse model by activation of the PI3K/Akt/eNOS signaling pathway [5]. In this study, decoction-mediated decreases in IL-6 and IL-1β in mice with ELG excision were examined.

Traditional medicinal herbs comprise several primarily botanical ingredients. The formulas are oral delivery forms of teas or decoctions. Some frequently used formulas are premade patent formulas. The traditional medicinal herbs in this study containing flavonoids, phenolic acids, and alkaloids are associated with an anti-inflammation and antioxidant effect in several disease models and may be a potential protection therapeutic strategy for DED [6,7,8]. However, the compounds in the decoction that act as therapeutic agents are currently unknown. Additionally, the dose of traditional medicinal herbs for long-term treatment is a challenge. Some herbs contain aristolochic acid and alkaloids, which have been related to stroke, nephropathy, and heart attack [37,38]. The inhibition of cell proliferation, death, and growth is essential for evaluating cytotoxic elements.

## 5. Conclusions

In this study, we evaluate the effect of the decoction of five traditional medicines: *Lycium chinense*, *Cuscuta chinensis*, *Senna tora*, *Ophiopogon japonicus*, and *Dendrobium nobile*, in a mouse model of dry eye and found that it was effective in suppression of ocular irritation and decrease in cytokine expression in the cornea. Our results indicate that the decoction exerted anti-inflammatory effects and rescued ocular defects. In the future, this traditional medicinal decoction may be used as a treatment for DED.

## Data Availability

All data generated or analyzed during this study are included in this article.
